# The relationship between transition shock and person-organization fit among newly graduated registered nurses in China: the mediating role of resilience

**DOI:** 10.1186/s12912-025-04118-1

**Published:** 2025-12-15

**Authors:** Liu Feng, Yutao Zeng, Xiaorong Chen, Yan Zhang, Juan Zhang

**Affiliations:** 1 School of Medicine, University of Electronic Science and Technology of China, Chengdu, 610072 China; 2 Department of Orthopaedics, Sichuan Provincial People’s Hospital, University of Electronic Science and Technology of China, Chengdu, 610072 China; 3 Department of Nursing, Sichuan Provincial People’s Hospital, University of Electronic Science and Technology of China, Chengdu, 610072 China

**Keywords:** Newly graduated registered nurse, Transition shock, Person-organization fit, Resilience, Mediating effect

## Abstract

**Background:**

A substantial body of research has been accumulated regarding the transition shock of newly graduated registered nurses (NGRNs). However, studies on person-organization fit remain insufficient. Improving person-organization fit among NGRNs can positively enhance job satisfaction, reduce turnover rates, and improve the quality of care. As a psychological resource, resilience can mitigate the negative impacts of job demands such as transition shock. However, the associative mechanisms among transition shock, person-organization fit, and resilience in NGRNs remain unclear.

**Methods:**

A cross-sectional survey was administered to 429 NGRNs recruited from three tertiary hospitals in Sichuan Province, China, between December 2024 and February 2025. Data were collected via an online platform using three validated self-report instruments: the Nurse Person-Organization Fit Assessment Scale, the Chinese version of the 10-item Connor-Davidson Resilience Scale (CD-RISC-10), and the Transition Shock of Newly Graduated Nurses scale. Statistical analyses were performed using IBM SPSS 24.0 for descriptive and correlational analyses. Structural equation modeling (SEM) was employed to test the hypothesized mediation model linking transition shock, resilience, and person-organization fit, with AMOS 24.0 used to estimate path coefficients and assess model fit.

**Results:**

The person-organization fit, transition shock, and resilience of NGRNs were at a moderate level or above. Person-organization fit was negatively correlated with transition shock (*r* = −0.417, *p* < 0.001), and positively correlated with resilience (*r* = 0.440, *p* < 0.001). Resilience was negatively correlated with transition shock (*r* = −0.332, *p* < 0.001). Psychometric testing confirmed excellent reliability and validity for most scales, while transition shock showed moderate indices, attributed to its complex multidimensional nature. The structural equation modeling (SEM) showed a satisfactory model fit: χ2/df = 3.004, comparative fit index (CFI) = 0.970, Tucker-Lewis index (TLI) = 0.953, root mean square error of approximation (RMSEA) = 0.068, standardized root mean square residual (SRMR) = 0.050. The mediating effect value of resilience is −0.127, which accounts for 24.5% of the total effect value of −0.518. Resilience partially mediates the relationship between NGRNs’ transition shock and person-organization fit.

**Conclusion:**

Transition shock directly and indirectly (via resilience) negatively impacts NGRNs’ person-organization fit, supporting the JD-R model in nursing transitions. Nursing managers should pay attention to the resilience level of NGRNs, implement dynamic assessments and time-sensitive intervention strategies to mitigate the multifaceted impacts of transition shock, foster enhancement of person-organization congruence, and consequently elevate job satisfaction, improve care quality, and reduce turnover rates.

## Introduction

*The State of the World’s Nursing 2025 Report* indicates that although the global nursing workforce continues to grow, reaching 29.8 million, the global “shortage” of nursing staff still amounts to 5.8 million [1]. This shortage, coupled with high nurse turnover, has become a protracted global issue [2]. The youngest generation of nurses are proven to be the most willing to leave their positions and the nursing profession [2]. It is reported that the turnover rate of newly graduated registered nurses (NGRNs) globally ranges from 25% to 70% [3, 4], while in China, the 1-year turnover rate among NGRNs is as high as 71.8% [5]. Consequently, retaining NGRNs has become an urgent priority amidst global workforce shortages, aging societies, and escalating healthcare demands [6].

This retention crisis is closely linked to the unique and daunting professional challenges that NGRNs encounter [7]. In China, generally speaking, nurses who have graduated within 2 years are called NGRNs [8]. The complexity of nursing and role requirements have exponentially increased nowadays. During the transition from academic settings to professional clinical practice, NGRNs encounter numerous pressures and challenges, including surging workloads, work environment, occupational stress, colleague relationships, feelings of incompetence, and low self-confidence [7, 9], making them particularly vulnerable to transition shock [10, 11]. Duchscher defined this phenomenon as a state of feeling confused, doubtful, disoriented, ambiguous, unsettled, or dissonant [12]. This not only reduces NGRNs’ work enthusiasm and job readiness [13] but also undermines their confidence, increases their tendency to self-blame [6], and impairs their work capabilities [14], making it difficult for them to meet organizational requirements and even leading to the occurrence of patient adverse events [15] or turnover [16].

Furthermore, in studies on nursing staff, existing research has shown that leadership style [17], cultural values [18], and professional growth opportunities [19], among other factors, are all associated with person-organization fit. This result can also be generalized to NGRNs. Person-organization fit, also known as person-organization matching, primarily describes the degree of congruence, complementarity, or correspondence between individuals and their affiliated organizations in terms of culture, values, goal pursuit, knowledge, and capabilities [17]. Relevant study [20] has also confirmed that there is a negative correlation between person-organization fit and transition shock among NGRNs. Poor fit leads to decreased job satisfaction and turnover intention [2, 9]. In contrast, higher person-organization fit is associated with improved nursing quality [21, 22], increased innovative behaviors [23], reduced anxiety/depression [24], and stronger organizational commitment [22] and retention intention [23], with job satisfaction also significantly enhanced [21, 25].

The Job Demands-Resources (JD-R) model [26] offers a robust theoretical framework for understanding these dynamics [27], positing that all occupational contexts comprise both job demands and job resources [8]. Job demands, such as transition shock, require sustained physical or mental effort—processes that may deplete energy and potentially lead to burnout. This state of exhaustion can, in turn, negatively color nurses’ perceptions of their workplace, making them feel a poorer fit with the organization’s values and goals. By contrast, job resources mitigate demands and their associated costs, while facilitating goal achievement and personal growth. Notably, the model extends beyond external job attributes to include internal personal resources, which collectively shape employees’ psychological and behavioral outcomes. Within this framework, we propose resilience as a critical personal resource [27, 28]: it buffers against transition shock and enables adaptive responses to workplace challenges.

As a quintessential personal resource within the JD-R framework, resilience refers to the capacity to adapt successfully to adversity, stress, or changing environmental demands [29]. This positive psychological construct is not static but a long-term, strategic, and dynamic process [30] that strengthens over time [31], acting as a protective factor against burnout [3] and a catalyst for clinical competence among NGRNs [32]. Empirical evidence shows that when NGRNs encounter transition shock, resilience enables active adjustment to stressful situations, thereby mitigating stress-related impairments [27]. Scholars emphasize that nurturing nurse resilience—especially among juniors—can enhance retention, productivity, and stress-coping capabilities [33]. Theoretically, resilience in the JD-R model serves a dual role: it mediates the impact of job demands (e.g., transition shock) on person-organization fit, both buffering strain and driving positive outcomes via its motivational properties.

Although existing research has confirmed the importance of person-organization fit in improving nurse retention and reducing burnout [2, 16, 34], further investigation is needed to clarify several key mechanisms within the Job Demands-Resources (JD-R) framework, especially in the context of Chinese NGRNs. Specifically, this study focuses on three aspects that have yet to be fully elucidated: (1) how transition shock, as a central job demand, may directly impede the development of person-organization fit; (2) whether resilience, as a critical personal resource, mediates this relationship by enhancing adaptation and mitigating negative outcomes; and (3) how job demands and personal resources interact dynamically to influence perceived fit. Therefore, this study applies the JD-R model to examine the mediating role of resilience in the relationship between transition shock and person-organization fit among NGRNs in China. The following hypotheses are proposed:

### Hypothesis 1

Transition shock is negatively correlated with resilience.

### Hypothesis 2

Resilience is positively correlated with person-organization fit.

### Hypothesis 3

There is a direct negative relationship between transition shock and person-organization fit.

### Hypothesis 4

Resilience mediates the relationship between transition shock and person-organization fit.

## Methods

### Study design

A cross-sectional research design was utilized in this study.

### Participants and setting

From December 2024 to February 2025, we conducted a cross-sectional study in three tertiary general hospitals across Sichuan Province, China. They were a provincial-level hospital, a specialized hospital, and a municipal-level hospital, with the sample size ratio being 2:1:1. Convenience sampling was used to recruit the NGRNs with an employment period ranging from 3 to 24 months in the three hospitals and a valid nurse practice qualification certificate. They were full-time nurses from the emergency department, the obstetrics and gynecology ward, the pediatric ward, the operating room, the Intensive Care Unit (ICU), the internal medicine ward, and the surgical ward, etc., and were willing to participate in the study. Those who were nursing students, further study nurses, or absent from work were excluded. Criteria for discontinuing participation in the study were as follows: 1) did not complete all investigations; (2) voluntary withdrawal of informed consent during the study.

Our sample size of 429 exceeds the recommended threshold for testing mediation effects with adequate power. With reference to the methodological guidelines of Fritz and MacKinnon [35], this sample size provides power greater than 0.8 for detecting indirect effects of small-to-medium magnitude, which is a realistic and conservative expectation for our research context.

### Measures

#### Demographic characteristics

We designed a questionnaire with the demographic characteristics of NGRNs by reviewing the literature and questioning experts, which included demographics (gender, age, education level, native of the province, only child, marital status), educational background (nursing as the first choice in university, receiving scholarships during university), work context (length of employment, hospital level, department, number of night shifts per month), lifestyle (daily sleep hours), and satisfaction measures (with the nursing profession, current department, and current income).

#### Person-Organization Fit

Xiang et al. [36] developed the Nurse Person-Organization Fit Assessment Scale in 2019, which consists of three dimensions: value fit, personal needs and organizational supply, and personal abilities and organizational demands, totaling 40 items. The scoring uses a Likert 5-point scale, where 1 point represents “very unimportant” and 5 points represents “very important”. The total score ranges from 40 to 200, with higher scores indicating a better fit. The overall Cronbach’s α coefficient of the scale is 0.986. In our study, the Cronbach’s alpha coefficient of each subscale ranged from 0.896 to 0.954.

#### Transition shock

The Transition Shock of Newly Graduated Nurses scale was developed by Xue [37] based on the conceptual framework of the Transition Shock Theory and contains 27 items in four dimensions: physical, psychological, knowledge and skills, and sociocultural and developmental. A 5-point Likert scale was used, ranging from 1 (“completely inconsistent”) to 5 (“completely consistent”) with intermediate levels, with a total score of 27 to 135; higher scores indicate stronger transition shock. The Cronbach’s alpha coefficient of the four subscales of the original Chinese scale was in the range of 0.864–0.940, respectively [37]. In this study, the Cronbach’s alpha coefficient of each subscale ranged from 0.875 to 0.923.

#### Resilience

The 10-item Connor-Davidson Resilience Scale (CD-RISC-10) was extracted from the 25-item Connor-Davidson Resilience Scale (CD-RISC-25) by Campbell-Sills and Stein [38], using a Likert 5-point scoring method: never-0, rarely-1, sometimes-2, often-3, always-4. The total score ranges from 0 to 40. A higher total score indicates a higher level of resilience. Ye et al. [39] translated and revised the original scale into Chinese, with a Cronbach’s α coefficient of 0.877. In this study, the Cronbach’s α coefficient was 0.933.

### Ethical consideration

This study was conducted in accordance with the Declaration of Helsinki and received ethical approval from the authors’ institution. Detailed information regarding ethics approval and consent is provided in the “Declarations” section at the end of this manuscript.

### Data collection

Prior to initiating the study, administrative consent was secured from all three participating hospitals. Three research assistants, each designated from the nursing departments’ training units, were recruited to facilitate participant enrollment. A detailed explanation was provided to familiarize assistants with study objectives, participant eligibility criteria, and standardized data collection protocols.

Data collection employed an online survey approach. Unique QR codes linked to encrypted survey platforms were generated, rigorously tested for functionality, and distributed exclusively to the research assistants. These assistants were tasked with identifying potential participants, providing oral and written explanations of the study’s purpose, and emphasizing voluntary participation and data anonymity. Eligible candidates were instructed to independently scan the QR code using their personal smartphones, whereupon they were required to digitally sign an informed consent form before accessing the questionnaire.

To ensure data integrity, responses were automatically screened for completion time (< 5 min) and response pattern irregularities (e.g., straight-lining). Of the 468 questionnaires returned, 39 were excluded due to incomplete data or invalid response patterns, yielding 429 valid responses for analysis (effective response rate: 91.67%).

### Data analysis

Statistical analyses were performed utilizing IBM SPSS Statistics 24.0 and AMOS 24.0. All tests were two-tailed with a significance level set at *p* < 0.05.

#### Analytical strategy

Structural equation modeling (SEM) was selected as the primary analytical method for this study based on its specific advantages for testing our hypothesized model: (1) its capacity to simultaneously examine all hypothesized direct and indirect pathways among variables within a unified model; (2) its ability to model our core constructs as latent variables, thereby accounting for measurement error and providing more accurate parameter estimates; and (3) the robustness of bootstrapping procedures within SEM for testing mediation effects, which does not rely on normal distribution assumptions [40]. Furthermore, SEM provides a suite of goodness-of-fit indices for a comprehensive evaluation of the proposed theoretical model.

#### Preliminary and descriptive analyses

Categorical variables were reported as frequencies and percentages, while continuous variables meeting normality assumptions were presented as mean ± standard deviation (*M* ± *SD*). Bivariate correlations among person-organization fit, transition shock, and resilience were examined using Pearson’s product-moment correlation coefficient. Internal consistency reliability of the measurement scales was evaluated via Cronbach’s α coefficient.

#### Testing of hypotheses via SEM

Hypotheses were tested using a two-step structural equation modeling approach. Step 1: Measurement Model. Confirmatory factor analysis (CFA) was conducted to assess the construct validity of the measurement model. Model fit was evaluated using multiple indices: comparative fit index (CFI), Tucker-Lewis index (TLI), root mean square error of approximation (RMSEA), and standardized root mean square residual (SRMR). Additionally, we examined standardized factor loadings, composite reliability (CR), and average variance extracted (AVE) to assess the reliability and convergent validity of the latent variables. Step 2: Structural Model. The direct and mediating effects were estimated using bias-corrected bootstrap resampling with 2000 iterations and a 95% confidence interval. This method was employed to test the hypothesized mediation model, with resilience posited as a mediator between transition shock and person-organization fit.

## Results

### Descriptive statistics of socio-demographic information

A total of 429 NGRNs, 73 (17.02%) male and 356 (82.98%) female, were included in this study. Their ages ranged from 20 to 26. In terms of educational level, 35 (8.16%) and 394 (91.84%) participants had a specialist, bachelor’s degree or above, respectively (Table 1).

**Table 1:** Sociodemographic information of NGRNs (*n* = 429)

Variable	Category	*n*	%
Gender	Male	73	17.02
	Female	356	82.98
Age(years)	20	7	1.63
	21	26	6.06
	22	41	9.56
	23	105	24.48
	24	145	33.80
	25	92	21.45
	26	13	3.03
First choice in college	Nursing	256	59.67
	Not Nursing	173	40.33
Educational level	Specialist	35	8.16
	Bachelor’s degree or above	394	91.84
Scholarship in college	Yes	280	65.27
	No	149	34.73
Married	Yes	7	1.63
	No	422	98.37
Only-child generation	Yes	176	41.03
	No	253	58.97
Natives of this province	Yes	341	79.49
	No	88	20.51
Length of employment(years)	< 1	179	41.72
	>=1	250	58.28
Hospital level	Provincial hospital	196	45.69
	Specialized hospital	120	27.97
	Municipal hospital	113	26.34
Department	Internal Medicine	110	25.64
	Surgery	142	33.10
	Obstetrics and Gynecology	27	6.29
	Pediatrics	19	4.43
	Operating room	44	10.26
	Outpatient/Emergency department	24	5.59
	ICU	42	9.79
	Other	21	4.90
Night shift frequency per month	0	17	3.96
	1–2	37	8.62
	3–4	150	34.97
	5–8	143	33.33
	> 8	82	19.11
Sleep duration per day	< 4 h	22	5.13
	4 to < 6 h	130	30.30
	6 to 8 h	239	55.71
	> 8 h	38	8.86
Satisfaction with the nursing profession	Very satisfied	80	18.65
	Generally satisfied	287	66.90
	Dissatisfied	45	10.49
	Very dissatisfied	17	3.96
Satisfaction with the current department	Very satisfied	170	39.63
	Generally satisfied	228	53.15
	Dissatisfied	24	5.59
	Very dissatisfied	7	1.63
Satisfaction with current income	Very satisfied	48	11.19
	Generally satisfied	172	40.09
	Dissatisfied	131	30.54
	Very dissatisfied	78	18.18

Note: ICU = Intensive Care Unit

### Descriptive statistics of transition shock, resilience, and person-organization fit

The overall person-organization fit score indicated a moderately high level of perceived fit (*M* ± *SD*: 161.15 ± 30.76; Item Score: 4.03 ± 0.77). Transition shock was reported at a moderate overall level (83.37 ± 18.21). Examination of its subdimensions revealed variation, with the highest scores in the physical domain and the lowest in the sociocultural and developmental domain. Resilience levels also were in the moderate range (26.14 ± 7.73) (Table 2).

**Table 2:** Scores of person-organization fit, transition shock, and resilience (*M* ± *SD*, *n* = 429)

Variable	Range	Item Score	Total Score
**Person-Organization Fit**	42–199	4.03 ± 0.77	161.15 ± 30.76
Value Fit	23–110	4.00 ± 0.79	87.96 ± 17.47
Personal Needs-Organizational Supply	10–50	4.06 ± 0.92	40.63 ± 9.18
Personal Ability-Organizational Demand	9–40	4.07 ± 0.85	32.57 ± 6.76
**Transition shock**	27–135	3.09 ± 0.67	83.37 ± 18.21
Physical	6–30	3.38 ± 1.02	20.27 ± 6.15
Psychological	8–40	3.32 ± 1.02	26.54 ± 8.17
Knowledge and skills	5–25	3.07 ± 0.98	15.34 ± 4.92
Sociocultural and developmental	8–40	2.65 ± 0.92	21.22 ± 7.37
**Resilience**	0–40	2.61 ± 0.77	26.14 ± 7.73

Note: *M* = Mean; *SD* = Standard Deviation

### Correlations among transition shock, resilience, and person-organization fit

Pearson correlation analysis revealed significant relationships among the key variables, all at the *p* < 0.01 level. As hypothesized, a significant negative correlation of moderate strength was observed between transition shock and person-organization fit (*r* = −0.417). Conversely, resilience demonstrated a significant positive correlation of moderate strength with person-organization fit (*r* = 0.440). Furthermore, resilience was significantly negatively correlated with transition shock (*r* = −0.332), indicating that higher levels of transition shock were associated with lower resilience (Table 3).

**Table 3:** Correlations among transition shock, resilience, and person-organization fit (*r*, *n* = 429)

Variable	(1)	(2)	(3)	(4)	(5)	(6)	(7)	(8)	(9)	(10)
(1) **TS**	1									
(2) TS1	0.653^***^	1								
(3) TS2	0.671^***^	0.156^**^	1							
(4) TS3	0.662^***^	0.337^***^	0.278^***^	1						
(5) TS4	0.742^***^	0.384^***^	0.235^***^	0.381^***^	1					
(6) **Resilience**	−0.332^***^	−0.194^***^	−0.233^***^	−0.272^***^	−0.219^***^	1				
(7) **POF**	−0.417^***^	−0.202^***^	−0.352^***^	−0.332^***^	−0.251^***^	0.440^***^	1			
(8) POF1	−0.407^***^	−0.195^***^	−0.323^***^	−0.329^***^	−0.268^***^	0.406^***^	0.958^***^	1		
(9) POF2	−0.387^***^	−0.213^***^	−0.343^***^	−0.275^***^	−0.216^***^	0.377^***^	0.897^***^	0.766^***^	1	
(10) POF3	−0.319^***^	−0.125^**^	−0.301^***^	−0.290^***^	−0.157^**^	0.442^***^	0.858^***^	0.733^***^	0.743^***^	1

Notes: ***p<0.001;** *p*<0.01;TS = Transition shock, TS1 = Physical; TS2 = Psychological; TS3 = Knowledge and skills; TS4 = Sociocultural and developmental,

POF = Person-Organization Fit; POF1 = Value Fit; POF2 = Personal Needs-Organizational Supply; POF3 = Personal Ability-Organizational Demand

### Regression analysis of person-organization fit

The regression model significantly predicted person-organization fit among NGRNs (Adjusted *R²* = 0.297, *F* = 11.065, *p* < 0.001). As shown in Table 4, transition shock (*β* = −0.299, *p* < 0.001) and daily sleep duration (*β* = −0.146, *p* = 0.001) emerged as significant negative predictors of person-organization fit. In contrast, resilience was a significant positive predictor (*β* = 0.327, *p* < 0.001). Satisfaction with the current department also demonstrated a significant, though weaker, negative relationship with the outcome (*β* = −0.099, *p* = 0.049). Multicollinearity diagnostics indicated no cause for concern (all VIFs < 1.6).

**Table 4:** Regression analysis of person-organization fit. (*n* = 429)

Variable	B	SE	β	t	*p*	Tolerance	VIF
Constant	188.896	26.104		7.236	＜0.001		
Sleep duration per day	−6.358	1.907	−0.146	−3.334	0.001	0.859	1.165
Satisfaction with the current department	−4.665	2.364	−0.099	−1.974	0.049	0.656	1.524
Transition shock	−0.505	0.079	−0.299	−6.410	＜0.001	0.754	1.326
Resilience	1.302	0.177	0.327	7.337	＜0.001	0.825	1.211

Notes: SE = Standard Error; VIF = Variance Inflation Factor; *R*^*2*^ = 0.327; adjusted *R*^*2*^ = 0.297; *F* = 11.065; *p* < 0.001; The variable “Satisfaction with the current department” was reverse-scored for analysis and higher values represent lower satisfaction (i.e., higher dissatisfaction)

### Measurement model assessment

The confirmatory factor analysis results supported the construct validity of the measurement model (Table 5). All factor loadings were significant and of acceptable magnitude. The composite reliability values indicated satisfactory internal consistency. Convergent validity, as measured by the average variance extracted (AVE), was strong for person-organization fit but was below the recommended threshold of 0.5 for transition shock.

**Table 5:** Results of measurement model testing (Confirmatory factor Analysis)

Latent Variable	Manifest Variable	Standardized Factor Loadings	Estimate	*p*	CR	AVE
Transition Shock (TS)	TS1	0.514	0.148	< 0.001	0.636	0.308
TS2	0.446	0.198	< 0.001			
TS3	0.648	0.204	< 0.001			
TS4	0.592	0.550	< 0.001			
Person-Organization Fit (POF)	POF1	0.875	0.559	< 0.001	0.899	0.748
POF2	0.874	0.834	< 0.001			
POF3	0.845	0.770	< 0.001			

Notes: TS = Transition Shock; POF = Person-Organization Fit; CR = Composite Reliability; AVE = Average Variance Extracted. Resilience was modeled as an observed composite variable in the structural model and was not included in this CFA

### Test of the hypothesized model

**Fig. 1:**
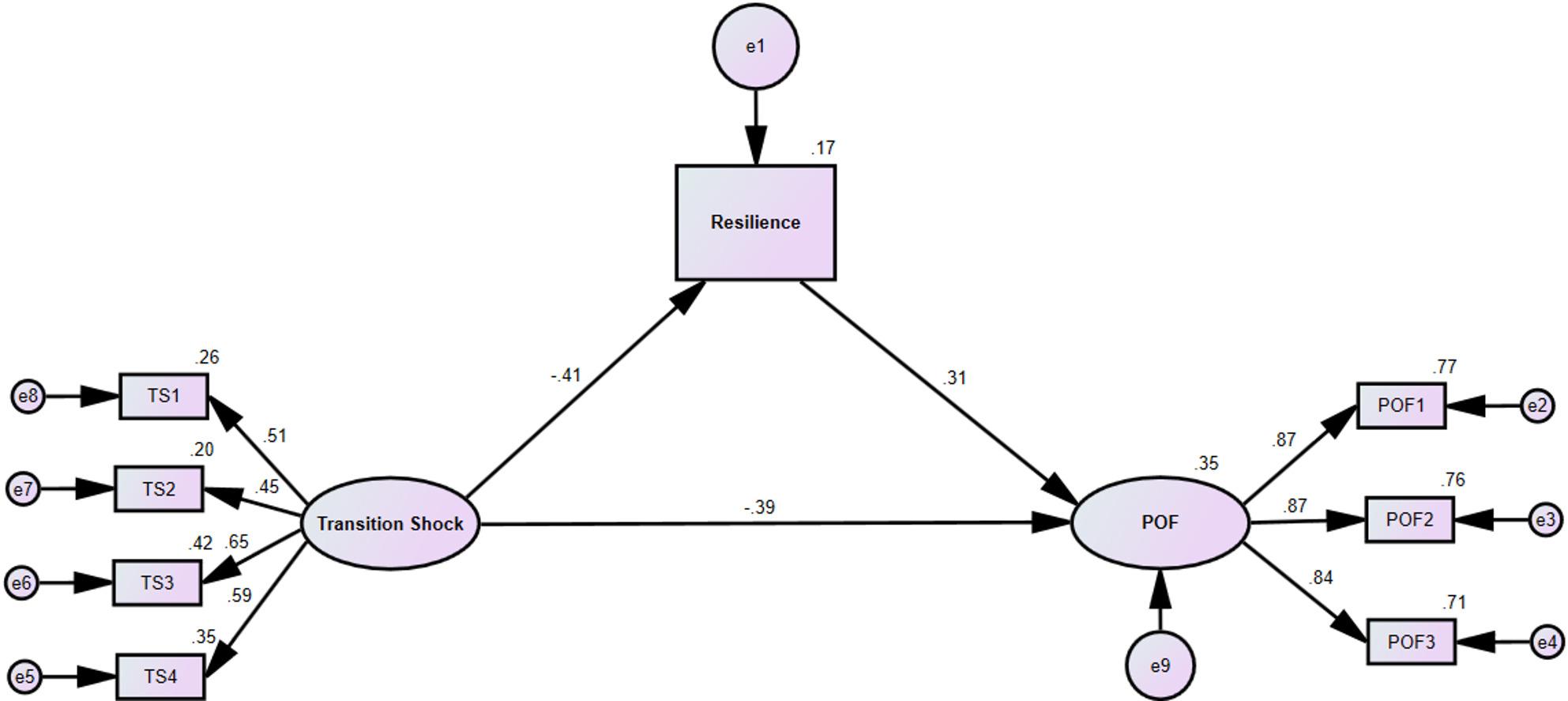
Path model shows mediating effects of resilience between transition shock and person-organization fit. Note: TS1 = Physical; TS2 = Psychological; TS3 = Knowledge and skills; TS4 = Sociocultural and developmental; POF = Person-Organization Fit; POF1 = Value Fit; POF2 = Personal Needs-Organizational Supply; POF3 = Personal Ability- Organizational Demand

The structural equation model demonstrated acceptable fit to the data: χ²/df = 3.004, comparative fit index (CFI) = 0.970, Tucker-Lewis index (TLI) = 0.953, root mean square error of approximation (RMSEA) = 0.068, standardized root mean square residual (SRMR) = 0.050.The path diagram and coefficients are shown in Fig. 1. Standardized path coefficients for all direct and indirect effects are presented in Table 6. The bootstrap analysis revealed a significant indirect effect of transition shock on person-organization fit through resilience (effect = −0.127, *p* < 0.001), accounting for 24.5% of the total effect (total effect = −0.518, *p* <0.001). The direct effect of transition shock on person-organization fit remained significant (effect = −0.391, *p* <0.001).

**Table 6:** Mediating effects of resilience between transition shock and person-organization fit (*n* = 429)

Effects	Model paths	Estimate	SE	*p*	95%CI	Effect ratio
Direct effects	TS→RL	−0.411	0.054	<0.001	[−0.511, −0.298]	
	RL→POF	0.308	0.054	<0.001	[0.195, 0.407]	
	TS→POF	−0.391	0.067	<0.001	[−0.522, −0.255]	75.5%
Indirect effects	TS→RL→POF	−0.127	0.025	<0.001	[−0.178, −0.083]	24.5%
Total effects		−0.518	0.058	<0.001	[−0.625, −0.392]	

Notes: SE = Standard Error; CI = Confidence Interval; TS = transition shock; RL = resilience; POF = person-organization fit

## Discussion

Utilizing the Job Demands-Resources (JD-R) model as a theoretical framework, this study offers a comprehensive empirical investigation into the mechanisms through which transition shock (TS) impacts the person-organization fit (POF) of NGRNs, with a specific focus on the mediating role of resilience. The findings provide robust support for our hypothesized model, revealing that resilience functions as a critical job resource that partially explains how the demanding experience of TS leads to poorer POF.

### Psychometric appraisal and methodological rigor

The reliability and validity of the reflective latent constructs (TS and POF) were first assessed. Confirmatory factor analysis (CFA) for these two constructs demonstrated that all factor loadings were statistically significant (*p* < 0.001) (see Table 5). POF construct exhibited high and robust loadings (0.845–0.875), high composite reliability (CR = 0.899), and excellent convergent validity (AVE = 0.748). Resilience, treated as an observed composite, also demonstrated high internal consistency (Cronbach’s α = 0.933).

Subsequently, the full structural equation model (incorporating both the measurement and the hypothesized structural paths) was tested. This model demonstrated an excellent fit to the data, which aligns with conventional thresholds for acceptable fit [41]. This good fit provides comprehensive support for the entire theoretical model, including the structural relationships and the measurement structure of the latent variables.

A detailed psychometric appraisal, however, offers a nuanced insight: the TS scale showed more moderate factor loadings (0.446–0.648). Its Composite Reliability (CR = 0.636) was acceptable, while its Average Variance Extracted (AVE = 0.308) was below the 0.5 threshold [42]. Rather than merely a limitation, this is a meaningful finding that underscores the complex, multifaceted nature of the TS construct itself. The items capture distinct yet interrelated challenges—physical exhaustion, psychological distress, skill deficits, and socio-cultural struggles—that together form a holistic latent variable. The moderate loadings and AVE suggest that no single dimension overwhelmingly defines the experience; it is the compound of these demands that constitutes the shock. Together, these findings collectively affirm the substantive theory and provide strong justification for employing the total scale score [43].

### Descriptive statistics and variable relationships

The study also showed that the total score of TS among NGRNs was 83.37 ± 18.21, which was at a moderate level and consistent with previous studies [44, 45]. The total score of resilience was 26.14 ± 7.73, at a moderate level, slightly lower than the previous study [46]. The POF score of NGRNs was marginally above moderate—higher than the score in Wang et al.‘s research [47]. The reason may be that their study was conducted during the COVID-19 pandemic (in October 2021), which might have imposed more work requirements (job demands) on NGRNs, and thus reducing their personal-organizational fit.

Correlation analysis revealed that TS was significantly negatively correlated with both resilience (*r* = −0.332) and POF (*r* = −0.417), while resilience was significantly positively correlated with POF (*r* = 0.440). This pattern aligns with empirical findings on the relationships between work stress, personal resources, and work adaptation [27, 48].

### Hypothesis testing and theoretical dialogue

Regression analysis, after controlling for covariates, confirmed that TS (*β* = −0.299) and resilience (*β* = 0.327) were the two strongest predictors of POF. Facing complex clinical situations and surging workloads, NGRNs feel confused, oppressed, and unconfident, which affects their work adaptability [49] and ability [14], fails to meet organizational requirements, and leads to dissatisfaction and alienation from the organization, resulting in a decrease in POF. The negative effect of TS validates the main effect hypothesis (H1) of this study and is also supported by Liu et al. [50], who found that resilience directly influences TS. The positive effect of resilience supports H2, extending the findings of Baek et al. [51] on the beneficial effect of resilience on career satisfaction to the important outcome variable of POF. Although there is no direct previous research supporting this point, existing studies have shown that resilience is positively correlated with career satisfaction [52], which is positively correlated with POF [53]. This indirectly demonstrates our results. TS’s direct negative effect on POF (*β* = −0.299; *β* = −0.391 in mediation analysis) validated H3, consistent with Wang et al. [47] on TS’s impact on new nurse outcomes.

Interestingly, the results showed that NGRNs’ POF was also significantly associated with their daily sleep duration and satisfaction with the current department. Longer self-reported daily sleep duration emerged as a predictor of lower POF (*β* = −0.146, *p* = 0.001), rather than a protective one. A plausible explanation for this counterintuitive result is that longer sleep duration may be a marker of daytime sleepiness. This sleepiness-driven social withdrawal, demonstrated by Holding et al. [54], directly exacerbates the social and cultural dimensions of TS by limiting engagement and acculturation, thereby leading to lower POF. Existing study [55] has linked severe sleep disorders in nurses to higher turnover from their first organization, highlighting the need to confirm the relationship between sleep and POF. Future studies could enhance precision by employing objective sleep quality measures (e.g., wearable devices [56]) rather than relying solely on self-reported duration. Furthermore, this study found that satisfaction with the department had a marginally significant negative predictive effect on POF (*β* = −0.099, *p* = 0.049). As the satisfaction scale was reverse-scored, this result demonstrates that higher departmental satisfaction predicts greater POF. This finding reflects the core concept of POF—that alignment with one’s immediate work environment strengthens the perception of shared values with the overall organization [18].

Most importantly, mediation analysis confirmed the partial mediating role of resilience (H4). This indicates that TS not only directly impairs POF (direct effect: *β* = −0.391) but also exerts an indirect effect by depleting resilience, a key personal resource (indirect effect: *β* = −0.127). This finding refines our understanding of the underlying mechanisms and engages in a constructive dialogue with the core proposition of the JD-R model [26]—that job demands impair well-being through an energy depletion process, while personal resources facilitate engagement through a motivational process. The results provide empirical evidence for the application of this theory to the transition period of NGRNs and partly explain the “black box” between TS and POF observed in previous studies [47].

### Research implications, limitations, and future directions

The main contribution of this study lies in the empirical testing and confirmation of the mediating mechanism of resilience between TS and POF, deepening the understanding of the role of “personal resources” in the JD-R model. The findings suggest that to promote organizational integration of NGRNs, healthcare institutions should adopt a dual strategy: First, reduce the intensity of TS at its source through measures such as transition programs [57], peer support [58]; Second, enhance nurses’ job resources by enhancing their resilience via stress management training or employee assistance programs (the Community Resiliency Model [9], mindfulness interventions [6], digital resilience interventions [59]) to enhance POF.

This study has several limitations that should be acknowledged. First, the cross-sectional design prevents causal inferences, and the sample from a single region in western China limits generalizability; future research should employ longitudinal designs and multi-region sampling to enhance validity. Second, reliance on self-report measures may introduce biases such as social desirability or recall errors. Finally, the fact that the identified mediation accounts for only 24.5% of the effect strongly suggests the existence of other important explanatory variables, warranting investigation in future studies.

## Conclusion

The results of this study demonstrate that transition shock negatively affects NGRNs’ person-organization fit both directly and indirectly through the mediating role of resilience. These findings not only corroborate the application of the Job Demands-Resources (JD-R) model within nursing transition contexts but also provide actionable insights for intervention. Consequently, we recommend that nursing administrators move beyond merely monitoring transition shock and prioritize the dynamic assessment of resilience. Implementing timely, resilience-building interventions is crucial to mitigate the negative impact of transition shock, enhance person-organization fit, and ultimately improve job satisfaction, care quality, and retention among NGRNs.

## Data Availability

The datasets used and/or analyzed during the current study are available from the corresponding author on reasonable request.
